# Antibacterial carbon dots

**DOI:** 10.1016/j.mtbio.2024.101383

**Published:** 2024-12-05

**Authors:** Shuaishuai Wang, Dapeng Wang, Guoliang Wang, Minglei Zhang, Yirong Sun, Jianxun Ding

**Affiliations:** a Department of Orthopedic Surgery, China-Japan Union Hospital of Jilin University, 126 Xiantai Street, Changchun 130033, PR China; b Key Laboratory of Polymer Ecomaterials, Changchun Institute of Applied Chemistry, Chinese Academy of Sciences, 5625 Renmin Street, Changchun 130022, PR China; c School of Mechanical and Aerospace Engineering, Jilin University, 5988 Renmin Street, Changchun 130033, PR China; d School of Applied Chemistry and Engineering, University of Science and Technology of China, 96 Jinzhai Road, Hefei 230026, PR China

**Keywords:** Carbon dot, Antibacterial performance, Physical destroy, Biochemical damage, Synergistic effect

## Abstract

Bacterial infections significantly threaten human health, leading to severe diseases and complications across multiple systems and organs. Antibiotics remain the primary treatment strategy for these infections. However, the growing resistance of bacteria to conventional antibiotics underscores the urgent need for safe and effective alternative treatments. In response, several approaches have been developed, including carbon dots (CDs), antimicrobial peptides, and antimicrobial polymers, all of which have proven effective in combating bacterial resistance. Among these, CDs stand out due to their unique advantages, including low preparation cost, stable physicochemical properties, high biocompatibility, tunable surface chemistry, strong photoluminescence, and efficient generation of reactive oxygen species. These features make CDs highly promising in antibacterial applications. This review explores the development of antibacterial CDs, focusing on their mechanisms of action—physical destroy, biochemical damage, and synergistic effects—while highlighting their potential for clinical use as antibacterial agents.

## Graphical abstract

**Figure:**
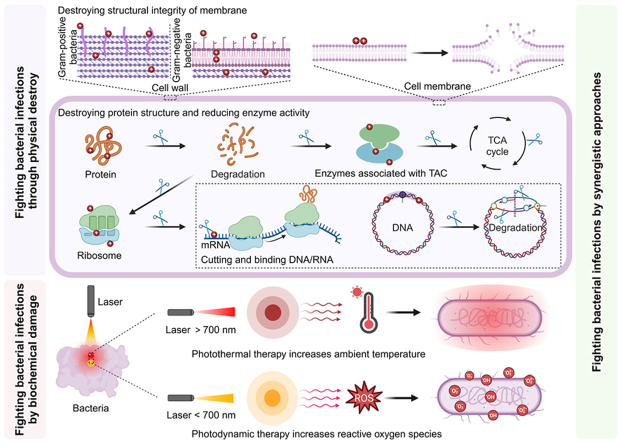

## 1 Introduction

Bacterial pathogens are the root cause of persistent and widespread infectious diseases, including pneumonia, osteomyelitis, and cellulitis, which continue to challenge global health efforts [1]. Serious conditions caused by bacterial infections, such as sepsis and meningitis, remain a significant concern [2,3]. Despite advances in medical technology, the prevalence of infectious diseases is rising [4,5]. The prevalence of infectious diseases is alarming, with millions of new cases reported each year, posing a heavy burden on individuals and healthcare [6,7]. During the recent COVID-19 pandemic, up to 16% of critically ill patients had bacterial co-infections [8]. The primary clinical strategy to treat bacterial infections involves antibiotics [9], yet bacterial resistance is increasing alarmingly, with multidrug-resistant bacterial infections affecting 34% of patients with cirrhosis globally [10]. Simultaneously, the number of newly developed antibiotics has declined [11]. Between 2017 and 2021, only 25 antibiotics reached phase III clinical trials or were approved by the Food and Drug Administration [12]. This situation underscores the urgent need for new antimicrobial agents and alternative approaches to combat bacterial infections.

Current research on antimicrobial substitutes focuses on bacteriophages, antimicrobial peptides, antimicrobial polymers, and antimicrobial carbon dots (CDs) [[13], [14], [15], [16]]. Phages specifically infect prokaryotic cells, whereas antimicrobial peptides exhibit a broad spectrum of antimicrobial properties, and both show the potential to address bacterial resistance [17,18]. However, their physical and chemical instability, along with complex production processes, hamper their practical application [19,20,21,22]. Antimicrobial polymers, while promising due to their multi-mode synergistic mechanisms, are still in the exploratory stage of understanding their antibacterial mechanisms, biocompatibility, and practical application [23,24,25]. In contrast, CDs exhibit excellent stability, superior optical properties, low cost, and a rich array of surface functional groups, positioning them as promising antibiotic alternatives [26,27,28].

CDs, a class of nanomaterials known for their significant fluorescence properties, were first reported in 2004 and later defined in 2006 as zero-dimensional carbon nanomaterials with diameters ranging from 2 to 10 nm [29,30]. CDs usually have a coreshell structure, with the core formed by a central carbon core (sp^2^/sp^3^ skeleton) in the form of graphite lattice or amorphous carbon, and the shell composed of rich functional groups or polymer chains [31]. Since 2011, the number of reports on CDs has grown exponentially [32]. CDs are categorized into four types—graphene quantum dots (GQDs), carbon quantum dots, carbon nanodots, and carbide polymer dots—based on their synthesis precursors, nanostructures, and properties [33,34]. CD synthesis occurs *via* two main routes: top-down and bottom-up [35]. The top-down method involves the localized organization of inorganic carbon to form zero-dimensional carbon nanoparticles, while the bottom-up method refers to the carbonation of small organic molecules into CDs [36]. Different synthesis precursors produce CDs with varying surface functional groups, which determine their antimicrobial properties, targeting, and selectivity [37,38]. CDs exhibit multiple antimicrobial mechanisms, making it difficult for bacteria to develop resistance, thus highlighting their potential as effective antimicrobial agents [39,40].

**Scheme 1:**
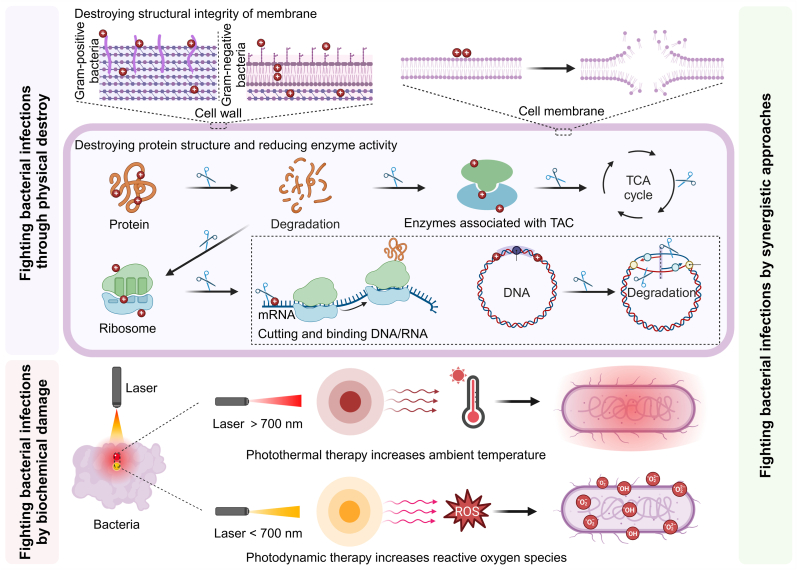
Antibacterial mechanisms of CDs. Created in BioRender. Y. Sun (2024) BioRender.com/w83c192.

Despite many reports on antimicrobial CDs, a comprehensive review of their mechanisms and antibacterial targets has been lacking. This review provides an in-depth analysis of the antimicrobial effects of CDs, examining three key mechanisms: physical destruction, biochemical damage, and synergistic effects (Scheme 1). It covers membrane disruption, interference with protein and enzyme function, DNA cleavage and binding, and bacterial elimination using photothermal therapy (PTT), photodynamic therapy (PDT), and synergistic treatments. We aim to clarify the antimicrobial mechanisms of CDs and inspire the design of advanced antimicrobial CD agents. The literature screening of this study was mainly based on PubMed, Web of Science, and Google Scholar databases.

## 2 Fighting bacterial infections through physical destroy

Disrupting bacterial structures to combat infections is an effective antimicrobial strategy [41,42]. Bacteria are unicellular organisms with key structural components, including the cell wall, cell membrane, cytoplasm, proteins, and nucleoid regions [43,44]. CDs with antimicrobial activity disrupt these structures, but different structures exhibit varying sensitivities to CDs [45].

### 2.1 Destroying structural integrity of membranes

The cell wall and cell membrane are two key bacterial structures with crucial roles in maintaining bacterial functions and cell morphology [46]. The bacterial cell wall is a rigid yet slightly flexible shell located outside the cell membrane, and its primary functions include maintaining bacterial shape, protecting bacteria from mechanical damage and osmotic pressure, and facilitating processes, such as bacterial recognition, adhesion, and flagellar colonization [43,47]. The composition and structure of the cell wall are essential for differentiating bacterial species and serve as key targets for bacterial destruction [48]. The major components of the bacterial cell wall include peptidoglycans, lipopolysaccharide (LPS) unique to Gram-negative bacteria (G^−^), and teichoic acid unique to Gram-positive bacteria (G^+^), which are arranged in a lattice-like structure to form a rigid shell [49].

G^+^ bacteria are generally more sensitive to lipophilic compounds than G^−^ bacteria, a difference attributed to the composition and structure of their cell walls. The peptidoglycan layer of G^+^ bacteria is typically thicker (20−80 nm) than that of G^−^ bacteria (10−20 nm) [50]. Tan and coworkers discovered that uncharged CDs did not impact bacterial growth, while positively charged CDs caused bacterial death by disrupting the membrane potential through electrostatic interactions between the bacteria and cations [45]. This occurs because the peptidoglycan layer of G^+^ bacteria contains a significant amount of negatively charged teichoic acid, which interacts with the cationic CDs, altering the cell wall's polarization state and disrupting its structure and function [51].

Wang et al. synthesized cationic CD using m-aminophenol and tartaric acid, which demonstrated antimicrobial efficiencies of more than 95.6% against G^+^ bacteria. In comparison, the same CD was only 22% effective against G^−^ bacteria at CD concentrations up to 250 μg mL^−1^ [52]. The accumulation of CDs on the bacterial surface leads to cell wall destruction [53,54]. Shangguan et al. examined the kinetics of liposome dye leakage in bacteria and found that cationic CDs significantly increased membrane fluidity in model membranes of *Escherichia coli* and dipalmitoyl phosphatidylcholine [55].

Although both G^+^ and G^−^ bacteria share similar cell wall components, the cell wall of G^−^ bacteria is more complex [43]. In addition to the peptidoglycan layer, the cell walls of G^−^ bacteria include an outer membrane consisting of a lipid bilayer and LPS, which is impermeable to macromolecules [47]. Compared with G^+^ bacteria, G^−^ bacteria were more tolerant to CDs, mainly due to the presence of an outer membrane. The outer membrane provides an additional physical barrier for CDs, which enhances their resistance to reactive oxygen species (ROS) and hinders the penetration of CDs [56]. LPS plays a significant role in stimulating mammal inflammation and is often called an endotoxin [57].

**Fig. 1:**
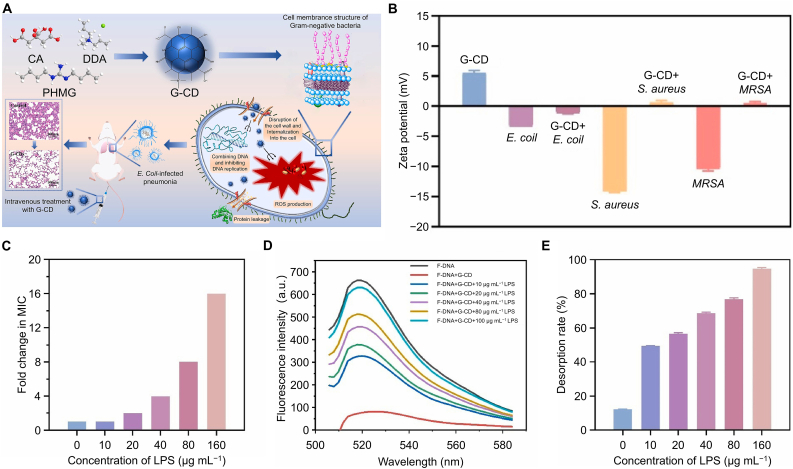
Antibacterial mechanism and effect of G-CD. (A) Synthetic route of antimicrobial carbon dot G-CD. (B) Zeta potential of G-CD and *E. coli* showed that G-CD interacted. (C) Addition of exogenous LPS to medium, competed with bacteria for binding G-CD and reduced the antibacterial properties of G-CD. (D) G-CD interacted with DNA, but its interaction with DNA was weaker than that of LPS. (E) With the increase of G-CD and LPS, the fluorescence of F-DNA gradually recovered, replacing the absorbed DNA. All statistical data are presented as the mean ± standard deviation (SD; *n* = 3). Reproduced with permission [59]. Copyright 2023, Elsevier Ltd.

Targeting LPS in G^−^ bacteria is a promising strategy for enhancing antimicrobial activity, as lipid A, a major component of LPS, contains anionic sites that bind to positively charged CDs [58]. Zhang et al. designed guanidinium CD (G-CD) that triggered bacterial death by specifically binding to lipid A, disrupting the outer membrane, and causing intracellular leakage [59]. *In vivo* experiments further confirmed the safety and therapeutic efficacy of G-CD against G^−^ bacteria (Fig. 1A). The zeta potential of G-CD was measured at +5.6 mV, indicating a positively charged surface, while the zeta potential of G^−^ bacteria confirmed their negative surface charge. The interaction of G-CD with bacterial surfaces was demonstrated through a positive shift in the zeta potential of *E. coli*, suggesting electrostatic attraction (Fig. 1B). Zhang et al. further evaluated the binding ability of G-CD to LPS by adding exogenous LPS to the culture medium (Fig. 1C). The exogenous LPS competed with the bacteria for G-CD binding, reducing the antimicrobial properties of G-CD. G-CD also interact with DNA, but this interaction is weaker compared to that with LPS, as F-DNA fluorescence gradually recovered upon the addition of LPS, which replaced the adsorbed DNA (Fig. 1D and E). Overall, G-CD demonstrate excellent antibacterial properties and selectivity, enabling efficient, targeted bacterial destruction.

The bacterial cell membrane, situated internal to the cell wall, is a flexible and semipermeable structure vital for maintaining cell integrity and regulating molecule transport [60]. Like the outer layers of G^−^ bacteria, the primary components of most bacterial membranes are phospholipid bilayers and proteins, which form the cell's fundamental framework [61]. Phospholipid bilayers possess hydrophilic and hydrophobic surfaces, allowing hydrophobic substances to penetrate the lipid layer of the membrane [62]. CDs with hydrophobic alkyl chains insert themselves into the phospholipid bilayer, enhancing their attachment to bacterial membranes and disrupting the plasma membrane's local structure [63].

Quaternized CDs synthesized by Zhao et al. from chitosan and its derivatives demonstrated broad-spectrum inhibitory effects against G^+^ and G^−^ bacteria [64]. The attachment of these CDs disrupted the electrical and physical balance of cell membrane, and in synergy with their positively charged and hydrophobic alkyl chains, they caused bacterial death [65,66]. This effect was particularly notable against *Staphylococcus aureus* biofilm at a concentration of 10.0 μg mL^−1^, which inhibited bacterial growth. Hydrophobic alkyl chains increased the binding affinity of CDs to cell membranes, leading to CD aggregation and disruption of membrane homeostasis [67,68]. However, this raises concerns about potential toxicity and nanoparticle aggregation, which may limit the practical application of quaternized CDs.

### 2.2 Destroying protein structure and reducing enzyme activity

Proteins are crucial for many biological processes in bacteria, playing essential roles in cell functions [69,70,71]. Targeting and disrupting bacterial proteins reduce bacterial activity, ultimately leading to cell death [72,73]. Recent studies have demonstrated that CDs interfere with bacterial protein synthesis, disrupt protein structures, and affect various proteins, including ribosomal proteins, structural proteins, extracellular proteins, and enzymes involved in metabolism [64,74,75].

Ribosomal proteins, vital for protein synthesis and gene expression regulation, are key targets of CDs [76]. Zhao et al. designed broad-spectrum antimicrobial CD with different targets in G^+^ and G^−^ bacteria [77]. In G^+^ bacteria, this CD exerts antibacterial effects by disrupting ribosomal function and inhibiting transcription and translation, similar to traditional antibiotics [78]. Additionally, the CD up-regulates proteins associated with RNA degradation, interfering with protein synthesis, post-translational modifications, and protein degradation in bacterial cells [77]. In G^−^ bacteria, CDs down-regulate various metabolism-related proteins, particularly those involved in the citrate cycle, ultimately disrupting cell respiration [79].

CDs also achieve antibacterial effects by disrupting structural proteins that maintain cell morphology and stability [80]. Xiao et al. used circular dichroism spectroscopy to study the interaction between CD and proteins extracted from *Staphylococcus aureus* [64]. Without CD, proteins generated a broad absorption peak at around 220 nm due to the n→π∗ jump in the α-helix structure. After co-incubation with CD, the absorption signal was diminished, indicating that CD disrupted the proteins' α-helical structures, thereby impairing bacterial activity [81]. Additionally, new protein absorption peaks were detected at 202 and 203 nm, suggesting that CD altered protein structural features [64,74].

Enzymes, as critical biocatalysts, facilitate various biological processes, including energy production, metabolic pathways, and DNA repair mechanisms [82,83]. *N*-acetylglucosamine acetyltransferase (NAG), a zinc-containing metalloenzyme, catalyzes a key step in lipid A biosynthesis, which is essential for the outer membrane of G^−^ bacteria [84]. Wang et al. prepared Artemisia annua leaf-derived CD, which significantly inhibited NAG activity by altering the enzyme's secondary structure [85]. The α-helix peak at 223 nm in NAG was more pronounced when CD was incorporated, indicating structural changes. The increased size distribution of NAG-CD complexes, as demonstrated by dynamic light scattering (DLS), suggested that CD bound to NAG, reducing their activity and disrupting lipid A biosynthesis [86]. This binding was further supported by decreased photoluminescence spectral intensity, indicating that CD interacted with NAG, ultimately leading to impaired bacterial biosynthesis [87].

Peptidoglycan is an essential constituent of the bacterial cell wall, providing a rigid constituent for growth, and an intact cell wall is the foundation of bacterial resistance to high internal osmotic pressure and external stimuli [88]. The peptidoglycan biosynthesis pathway, which occurs in multiple bacterial compartments, relies on amide ligases [89]. Qi et al. designed and synthesized bacterial affinity CD targeting amide ligase, which binds very tightly to bacteria and hides the activity of enzymes by vying with D-glutamate for attachment to amide ligase, thereby impeding the synthesis of bacterial walls [75]. Several other enzymes involved in bacterial genetic metabolism, such as DNA synthesis, *e.g.*, deoxyribonucleic acid synthesis-associated thionins, β-lactamases, and DNA gyrases, also show potential for CD inhibition [90,91].

In addition to intracellular proteins, CDs exhibit antimicrobial activity by altering the secondary structures of extracellular proteins [92]. Bacteria form biofilms by aggregating into microcolonies and secreting extracellular polymers [93]. Phenol-soluble modulin (PSM) are key components of extracellular polymers in G^+^ bacteria, playing a role in biofilm formation and dispersion, as well as being a major virulence determinant [94,95]. The self-assembly of PSM involves hydrogen bonding, hydrophobic interactions, π−π stacking, and van der Waals forces [96].

**Fig. 2:**
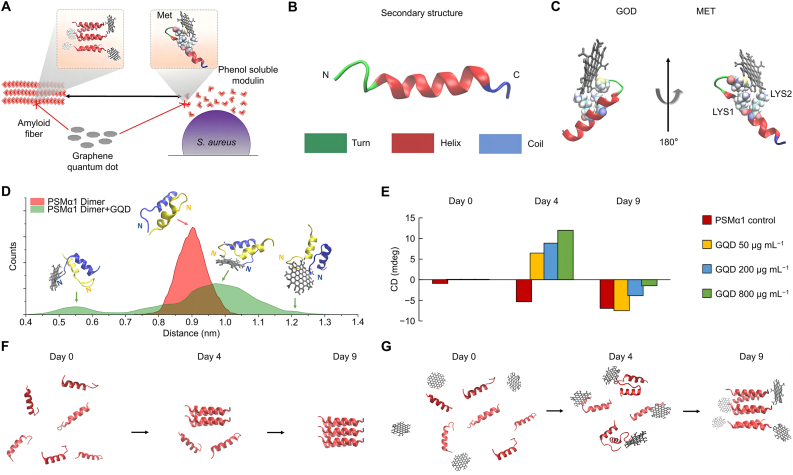
Antibacterial mechanism and effect of GQD. (A) Mimetic peptides bound biomolecules that bound GQD to PSMα1 to form supramolecular complexes. (B) Structure after interaction of GQD and PSM. (C) Formation of GQD conformational complex. (D) Dynamics of breakdown structure of GQD. (E) Concentration dependent secondary structure changes. (F,G) The interaction would affect the experimentally observed amyloid fibril formation. Reproduced with permission [97]. Copyright 2019, American Chemical Society.

To disrupt PSM assembly and interfere with biofilm maturation, VanEpps, Violi, and coworkers designed GQD that mimics peptide-binding biomolecules (Fig. 2A) [97]. Molecular dynamics simulations were conducted to investigate the interaction between GQD and PSMα1, a small α-helical amphiphilic peptide involved in biofilm formation. The PSMα1 peptide forms a solitary amphiphilic helix, slightly curved near its N- and C-termini (Fig. 2B). Positively charged residues near the N-terminus, such as methionine and lysine, participate in electrostatic interactions with −COO^−^ groups at the edge of GQD, forming a stable complex (Fig. 2C). Proximity of GQDs disrupted the interaction between the N- and C-termini of peptides, leading to a reduction in dimer stability (Fig. 2D). Additionally, GQD interaction with the peptide N-terminus altered the backbone contact angle, stretching a region near the N-terminus and interfering with amyloid fibril formation, as indicated by β-sheet signaling (Fig. 2E), due to docking of GQD near the peptide N-terminus, and changes in secondary structure (Fig. 2F and G). While the anti-biofilm properties of CDs are still under investigation, further engineering to enhance the binding of CDs to PSM opens new avenues for antibacterial therapy.

### 2.3 Cutting and binding DNA/RNA

DNA and RNA are vital biomolecules in bacterial cells [98]. Bacterial DNA is usually a double-stranded structure, consisting of a circular main strand and an auxiliary strand running in reverse parallel to it, and this double-stranded form helps to maintain DNA stability and the integrity of genetic information [99,100]. In the bacterial genome, genetic information is encoded in a series of base sequences called genes, which control various biochemical processes and cell functions [101,102,103].

Positively charged small molecules penetrating the bacterial cell membrane bind to intracellular DNA [59]. Once inside the cell, nanoscale CDs unwind the naked bacterial DNA structure, thereby hindering bacterial proliferation. For instance, Song et al. extracted CDs with a diameter of approximately 5.4 nm from cigarette smoke and found that these CDs perforated the bacterial cell membrane and spread into the cells [104]. Because prokaryotic DNA lacks protective histones, CDs easily adsorb onto it, unwinding the double helix and inhibiting bacterial replication [64]. In addition, some studies have further enhanced CD entry into bacterial cells by doping them with metals, achieving antibacterial effects by interfering with DNA replication [105].

**Fig. 3:**
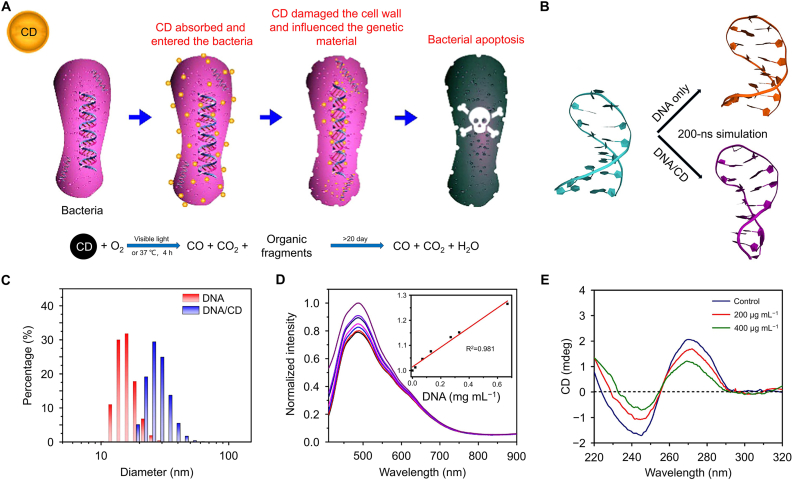
Antibacterial mechanism and effect of CD based on vitamin C. (A) Manufacture antibacterial CD from vitamin C. (B) Structure of CD-treated and non-CD-treated DNA. (C) Diameters of CD-treated and non-CD-treated DNA. (D) Fluorescence intensity of CD after addition of different concentrations of DNA. (E) Circular dichroism CD-treated and non-CD-treated DNA. Reproduced with permission [108]. Copyright 2018, American Chemical Society.

Cationic CDs interact with bacterial genomic DNA, preventing its amplification *via* PCR or replication [106,107]. For example, low-toxicity, degradable CD synthesized from vitamin C achieved a 100% bacterial mortality rate with increasing CD concentrations (Fig. 3A). DLS measurements demonstrated that DNA treated with CD exhibited a significant increase in diameter, indicating CD aggregation onto the DNA (Fig. 3B and C) [108]. The fluorescence patterns of CD after exposure to varying DNA concentrations showed a remarkable rise in emission intensity, suggesting that CD bound to DNA *via* non-covalent bonds (Fig. 3D). Additionally, CD interfered with the secondary structure of DNA. As seen in circular dichroism spectra, the intensities of both negative and positive bands were greatly reduced, confirming that CD loosened the DNA structure, leading to the unwinding of double helix (Fig. 3E). Molecular dynamics simulations revealed significant differences in the final confirmation of DNA between untreated and CD-treated groups. In the presence of CD, DNA hairpin structures exhibited unique distortions, including misaligning terminal base pairs. One study observed that a CD began attacking the terminal nucleobases of a DNA hairpin structure after 35.6 ns of interaction.

Genetic information is transcribed from DNA to RNA, translating this information into proteins [109]. CDs also interact with RNA, interfering with its role in protein synthesis. For example, CDs have been shown to bind with RNA and disrupt its ability to transmit information for translation, hindering protein production and bacterial growth [110]. Chatzimitakos et al. found that the sum of the absorption of CD and RNA at 260 nm was higher than that of the mixture of the two [111]. To determine the binding mode of CD and RNA, by exploring the quenching of CD in the presence and absence of two anionic substances, it was demonstrated that CD is embedded in the RNA structure and thus generates antimicrobial activity.

Bacterial communities that aggregate on surfaces form biofilms containing extracellular DNA (eDNA) [112]. The structure of eDNA in biofilm matrices is similar to Holliday junctions and acts as a cross-linker necessary for biofilm stabilization, which correlates positively with bacterial adhesion capacity [113]. Certain CDs inhibit bacterial biofilm growth by disrupting eDNA. For example, Pan et al. prepared Fe-doped CD that catalyzed the production of hydroxyl radical (·OH) from hydrogen peroxide (H_2_O_2_) and observed their destructive effects on *Staphylococcus aureus* and *Pseudomonas aeruginosa* biofilms [114]. Co-localization analyses with fluorescent nucleic acid dyes revealed a significant reduction in fluorescence signals within the biofilm, indicating eDNA degradation. Agarose gel electrophoresis further confirmed that the DNA levels in the CD-treated biofilms were greatly reduced, suggesting efficient eDNA cleavage was key to biofilm destabilization.

Overall, the structure and function of bacterial DNA and RNA are critical for cell activities, and CDs have shown significant potential in damaging bacterial DNA/RNA to inhibit cell growth or reproduction, providing promising applications in antibacterial therapies.

## 3 Fighting bacterial infections by biochemical damage

In addition to physically damaging key bacterial structures, CDs induce biochemical damage through external stimuli, such as photothermal and photodynamic effects [115]. Photothermal and photosensitivity therapies are novel, non-invasive treatment modalities that alter the physical and chemical microenvironments of local tissues *via* reactions of a photosensitizer at the infection site. However, the application of many photosensitizers in antimicrobial fields has been limited due to poor water solubility, low dispersibility, inefficient release of ROS, and high cytotoxicity [116].

Recently, CD-based photoinitiators have gained increasing research attention due to their favorable optical properties, wide range of sources, low cost, good biocompatibility, and numerous other advantages. Under light conditions, the photosensitizing CDs produce the photodynamic effect. That is, the electrons return from the excited state to the ground state accompanied by the generation of ROS [117]. Similarly, the photothermal agent CDs have a broadband absorption property, which absorbs light energy in the spectral range from visible light to near-infrared light and convert it into heat energy [118]. These characteristics make CDs a promising option for overcoming the limitations of conventional photoinitiators in antimicrobial therapy [55,119].

### 3.1 Photodynamic therapy increases reactive oxygen species

PDT is a highly effective and promising treatment developed to combat bacterial infections in response to increasing antibiotic resistance [120]. Similar to the photoexcited states and redox processes found in conventional nanoscale semiconductors, the CD-based PDT induces biochemical damage in bacteria through the generation of ROS by photosensitive molecules [117]. Certain CDs contain many unbound electrons in their initial state, which are excited to higher energy levels or singlet states when exposed to light at appropriate wavelengths [121]. These excited electrons undergo radiative relaxation, emitting fluorescence [122].

Photoactive compounds are activated by visible light, exciting hydrogen atoms or electrons within the photosensitizers to transfer energy and react with oxygen through type I (·OH, ·O_2_^−^, and H_2_O_2_) and type II (^1^O_2_) photosensitization mechanisms [123]. These processes generate ROS, such as singlet oxygen (^1^O_2_), ·OH, superoxide anion radical (·O_2_^−^), and H_2_O_2_, which are highly toxic to bacteria [124]. For example, ^1^O_2_ attacks and increases the porosity of bacterial cell walls, allowing free radicals to reach the membrane and induce lipid peroxidation [125].

Photosensitizing effects are not intrinsic to carbon nanodots, but doping nanodots with heteroatoms or metallic elements enhances these effects by inducing triplet properties through the heavy atom effect [126]. During the doping process, various heteroatoms and metallic elements—such as nitrogen, bromine, chlorine, zinc, and iron—are introduced into CDs to modulate their electronic structure [127,128]. This doping creates new defect sites that enhance photoluminescence intensity and facilitate functionalization [129]. In addition, the quantum yields of CDs are also closely related to their PDT effects. Quantum yields usually refer to the efficiencies of CDs to absorb light energy and convert it into fluorescence emission. This property directly affects the ability of CDs to produce ROS under photoexcitation conditions [130]. CDs with higher quantum yields more effectively excite ROS under light conditions, enhancing the antibacterial effect [131].

As a typical example, Huang et al. designed halogen/nitrogen-doped CD that exhibited bactericidal efficiencies of over 99% when exposed to white light LED irradiation (350 W m^−^^2^) for only 1 min [132]. Brominated CDs, as prepared by Knoblauch et al., were also used as unique photosensitizers for PDT [133]. Brominated CD generated ^1^O_2_ and ROS under laser irradiation *via* Type I and Type II photosensitization mechanisms, showing significant antimicrobial efficacy against G^−^ and G^+^ bacteria [132].

However, the efficiency of PDT in generating ROS in the anaerobic microenvironments is severely limited [134]. The continuous oxygen depletion caused by PDT worsens local hypoxia, creating a vicious cycle that hinders bacterial eradication [135]. Therefore, developing photodynamic therapies that alleviate localized hypoxia and enhance the aggregation-induced quenching effect of photosensitizers is crucial.

**Fig. 4:**
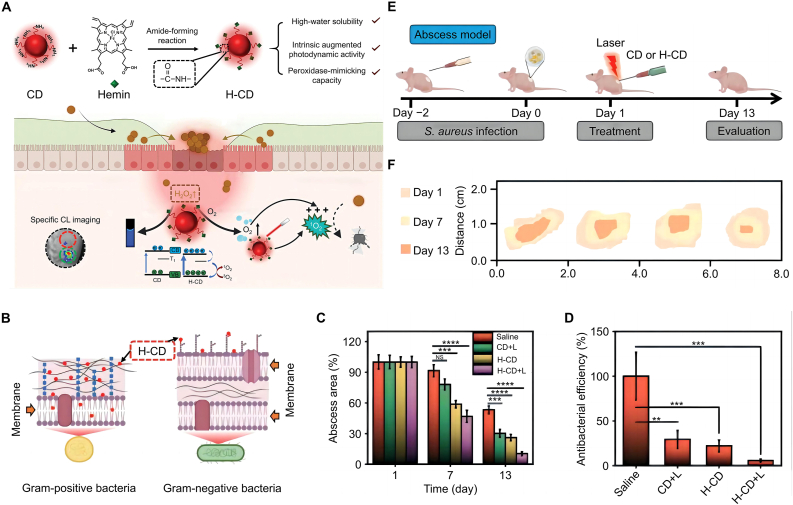
Antibacterial mechanism and effect of H-CD. (A) Synthesis method and antibacterial mechanism of H-CD. (B) Selective inactivation of G^+^ bacteria by H-CD. (C) CD showed an obvious bactericidal effect on all kinds of bacteria, and the colonies gradually decreased with the increase of treatment time. (D) Antibacterial efficacy of H-CD. (E) Therapeutic effects of H-CD on bacterial infection sites in rats. (F) Wound healing after H-CD treatment. All statistical data are represented as mean ± SD (*n* = 3; ∗*P* < 0.05, ∗∗*P* < 0.01, ∗∗∗*P* < 0.001, ∗∗∗∗*P* < 0.0001, NS represents no significant difference). Reproduced with permission [136]. Copyright 2023, John Wiley & Sons.

To address these challenges, Lin and coworkers synthesized heme-modified CD (H-CD) with enhanced photodynamic activity and oxygen self-supply for use in chemiluminescent imaging-guided PDT (Fig. 4A) [136]. H-CD selectively inactivated G^+^ bacteria under normal conditions, but when exposed to visible light, they showed great bactericidal effects against all bacterial strains (Fig. 4B and C). As CD concentration increased to 300 μg mL^−1^, antimicrobial efficiency approached 100% (Fig. 4D). The H-CD effectively treated bacterial infections in rats. When the CD entered the bacterial infection microenvironments, where H_2_O_2_ was significantly overexpressed, they triggered specific and long-lasting chemiluminescence, amplifying the effects of PDT (Fig. 4E and F).

### 3.2 Photothermal therapy increases ambient temperature

Bacterial metabolism depends heavily on the activity of internal enzymes, which is significantly diminished when ambient temperature exceeds their tolerance range. Under these conditions, bacterial reproduction is inhibited, making the bacteria more vulnerable to destruction [137]. CDs that convert light into heat increase ambient temperature when excited by near-infrared (NIR) light, leading to changes in the physical and chemical microenvironments inside and outside bacterial cells [118]. In this form of PTT, heat is generated by plasma resonance on the surface of CDs [138]. Nonradiative relaxation induces significant nanoscale molecular vibrations through electron-phonon coupling, which results in substantial heat generation on the macroscale [139].

Thus, CDs convert light into heat through photothermal conversion, causing local high temperatures at the infection sites that effectively destroy bacteria [140]. For instance, Chu et al. synthesized quaternary ammonium salt-modified NIR CDs with synergistic antibacterial properties under 808 nm irradiation [115]. These nanocomposites demonstrated excellent photothermal performance, with a photothermal conversion efficiency of approximately 35%, inhibiting the growth of both G^−^ and G^+^ bacteria by 99.5% and 99.8%, respectively.

PTT is a promising strategy for killing bacteria, especially for managing multi-drug-resistant bacterial infections [141]. However, prolonged exposure to excessively high temperatures may cause thermal damage to surrounding healthy tissues and potentially trigger new inflammation [142]. Therefore, it is crucial to minimize damage to normal tissues by using PTT at suitable temperatures and with high efficiency, which may require adding materials to improve the photothermal conversion efficiency [143].

**Fig. 5:**
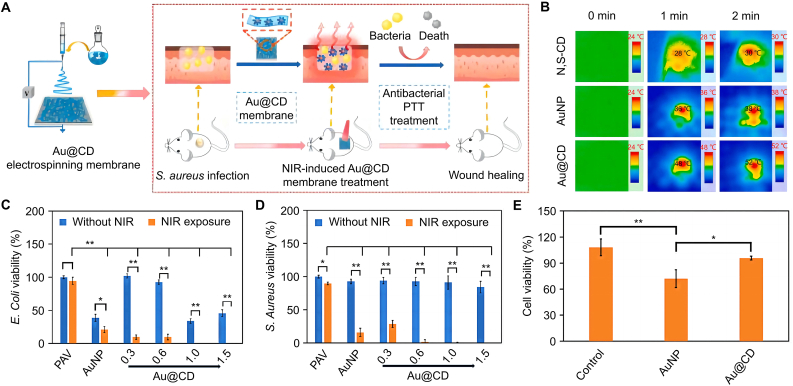
Antibacterial mechanism and effect of Au@CD. (A) Au@CD prepared by CD in combination with Au. (B) Wound thermal imaging of an infected model. (C) Viability of *E. coli* with and without NIR irradiation. (D) Survival of *S. aureus* with and without NIR irradiation. (E) Viability of cells with and without Au@CD. All statistical data are represented as mean ± SD (*n* = 3; *∗P* < 0.05, *∗∗P* < 0.01, *∗∗∗P* < 0.001, *∗∗∗∗P* < 0.0001, NS represents no significant difference). Reproduced with permission [146]. Copyright 2022, Elsevier Ltd.

Specific chemical and physical modifications redshift fluorescence emission peaks and enhance photothermal properties [144]. Introducing metal elements, such as zinc and sodium, into CDs enhances their ability to kill bacteria *via* photothermolysis [145]. These elements display localized surface plasmon resonances, resulting in intense excitation efficiency, making them ideal for bacterial eradication through PTT [144]. For example, Tian et al. prepared Au@CD by combining CD with gold (Au) (Fig. 5A) [146]. After visible light irradiation, the temperature of the Au@CD-containing solution was much higher than in other groups (Fig. 5B), indicating that binding CD to Au improved their photothermal conversion capability. Under NIR irradiation, bacterial inactivation significantly increased with higher Au@CD content, achieving 100% inactivation rates for two pathogenic bacteria (Fig. 5C−E).

In addition, introducing heteroatomic nitrogen further improves the photothermal conversion efficiency of CDs [147]. Geng et al. used highly graphitized nitrogen-doped CDs for PTT [148]. Nitrogen reactive sites function as paramagnetic hubs facilitating effective NIR absorption and conversion into photothermal energy [149]. These CDs exhibited strong NIR absorption in the 700−1200 nm range and demonstrated excellent antimicrobial and anti-biofilm activity against drug-resistant bacteria [148].

CDs-based PTT is a promising approach for enhancing antimicrobial efficacy, and it is less prone to traditional bacterial resistance mechanisms, such as increased drug metabolism and excretion. However, further research is required to improve antimicrobial efficiency, reduce the duration of antimicrobial time, and mitigate potential tissue damage from PTT. CD-based PTT in the NIR band, especially around 800 nm, has optimal tissue penetration and photothermal effects, effectively reducing water and hemoglobin absorption, thereby reducing damage to surrounding tissues [150]. Generally, 2–3 min of irradiation is sufficient to achieve an effective bactericidal or therapeutic effect without causing overheating or damage to surrounding healthy tissues [151,152]. To further reduce the risk of overheating, the treatment temperature can be precisely controlled by real-time temperature monitoring.

## 4 Fighting bacterial infections by synergistic approaches

Bacterial infections pose a significant health risk due to the overuse of antibiotics and the emergence of super-pathogenic bacteria. Solving this problem with single-modality antimicrobials based on CDs has proven challenging [153]. To overcome the limitations of single-mode antibacterials and enhance the effectiveness of CD-based therapies, it is essential to develop multiple antibacterial methods that work synergistically.

PTT alone often suffers from inadequate bactericidal efficiency because bacteria adapt to elevated temperatures by producing heat shock proteins, which increase their resistance to heat and have toxic effects on the host [154]. As a result, the required temperature for effective PTT may be higher, and the treatment time may be longer [155]. Additionally, the insufficient penetration depth of excitation light makes it difficult to treat deep tissue infections effectively [156]. High temperatures and prolonged irradiation in single-mode PTT treatments also disrupt the function of surrounding healthy cells [157].

In PDT, laser penetration generates ROS, which alters the microenvironments inside and outside the bacterial cells, disrupting their normal physiological functions [158]. However, large amounts of ROS are needed to combat bacteria effectively [159]. Excessive ROS damage the surrounding microenvironments, and the short lifespan of ROS makes it difficult to eradicate a sufficient number of bacteria when ROS production is inefficient [160]. Therefore, integrating PTT with PDT is necessary to enhance antimicrobial therapy.

**Fig. 6:**
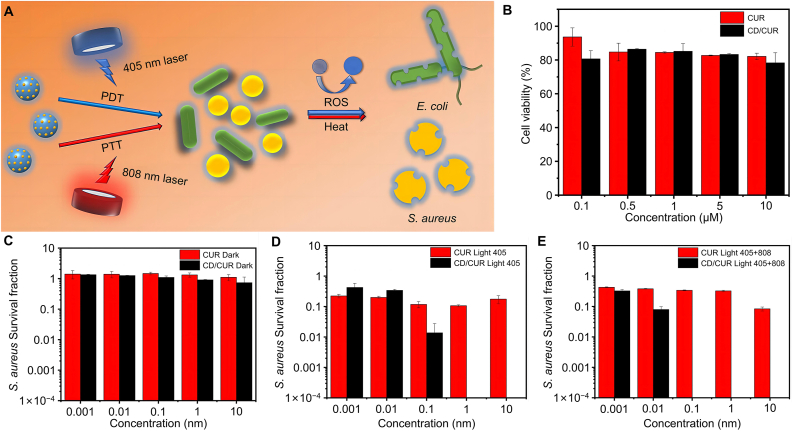
Antibacterial CD with synergistic PDT and PTT capabilities. (A) CD-based PDT and PTT combined antimicrobial therapy. (B) Comparison of cytocompatibility of different modalities. (C) Comparison of inhibitory effects of *S. aureus* without light. (D) Comparison of the inhibitory effects of *S. aureus* at 405 nm. (E) Comparison of the inhibitory effects of *S. aureus* at 405 and 808 nm. All statistical data are represented as mean ± SD (*n* = 3*; ∗P* < 0.05*, ∗∗P* < 0.01*, ∗∗∗P* < 0.001*,* ∗∗∗∗*P* < 0.0001*, NS* represents no significant difference). Reproduced with permission [161]. Copyright 2021, American Chemical Society.

Yan et al. synthesized antimicrobial CD synergizing PDT and PTT based on curcumin (CUR) (Fig. 6A) [161]. The limitations of CUR, as a conventional photosensitizer, can be compensated by the introduction of CD, and antimicrobial function could be improved by fluorescence resonance energy transfer in a compound nano photosensitizer system, where the high photothermal efficiency of CD can be efficiently converted under 808 nm NIR illumination. The water solubility and photostability of CUR in CD/CUR were improved under 405 nm NIR illumination, which was favorable for ROS production and enhanced the benefits of PDT [162]. Compared with the single-mode antimicrobial approach, such synergistic treatment, using low concentration, appropriate temperature, and moderate ROS, not only improved antimicrobial performance but also avoided damage to normal tissues (Fig. 6B). NIR light, with its high tissue penetration, increased the permeability of bacterial outer membranes and promoted ROS diffusion into cells during PDT, disrupting cell homeostasis and synergistically enhancing the antimicrobial effect (Fig. 6C−E).

To further enhance the antimicrobial efficiency of CDs, researchers have introduced quaternary ammonium compounds with hydrophobic alkyl chains, creating a PDT/PTT strategy [163]. This antimicrobial strategy involves synthesizing CD conjugated with quaternary ammonium compounds. The quaternary ammonium groups, along with the long hydrocarbon chains present on the surface of the CD, disrupt bacterial cell membranes, increasing their susceptibility to high temperatures and ROS [164]. Some studies have also utilized the negative charge of bacterial cells to adsorb positively charged CD. These CDs composite mediate bacterial membrane and cell wall damage through electrostatic interactions, enhancing the affinity of the composites for bacterial cells and enabling synergistic PDT/PTT antimicrobial effects [165,166].

Metal doping is another effective method for enhancing synergistic therapeutic effects. Zhou and his coworkers prepared ion-doped CD for the PDT/PTT treatment of bacterial infections [167]. The ROS generation efficiency of iron-doped CD under visible light was significantly improved, and their photothermal conversion efficiency increased by 35.11% compared to undoped metal CDs. In conclusion, CD-based synergistic therapies offer broad potential applications and significant research value in the fight against bacterial infections.

## 5 Conclusion and perspectives

At present, the clinical treatment of bacterial infections relies heavily on antibiotics. However, the increasing prevalence of bacterial resistance is making these infections more difficult to treat [10]. In recent years, extensive research into antibacterial CDs has led to the development of new CDs with diverse mechanisms and strategies for combating bacterial infections, as summarized in Table 1.

**Table 1:** Antimicrobial targets and mechanisms of CDs.

Raw material for CD	Antimicrobial mechanism	Antimicrobial target	MIC (μg mL^−1^)	Reference
Tartaric acid and aminophenol	Disruption of cell wall structure	Teichoic acid	50.0	[52]
Trimethoxysilane, trisodium citrate, and *N*-alkyl betaines		Teichoic acid	5.0	[54]
Citric acid, dime-thyldiallylammonium chloride, and polyhexamethyleneguanidine		Lipid A	2.5	[59]
Glycerol and dimethyloctadecyl [3-(trimethoxysilyl) propy] ammonium chloride	Disruption of cell membrane structure	Phospholipid bilayer	2.5	[171]
2,3-epoxypropyltrimethylammonium chloride and diallyldimethylammonium chloride	Disruption of protein structure and function	Ribosomal proteins	5.0	[77]
Artemisia argyi		Acetylglucosamine deacetylase	50.0	[85]
*O*-phenylenediamine and D-Glu		Amide ligase	100.0	[75]
Carbon fiber		Phenol soluble regulatory proteins	50.0	[97]
Cigarettes	Disruption of double helix structure of DNA	DNA	200.0	[104]
Citric acid and ethylenediamine			25.0	[107]
Chitosan, ferrous sulfate, heptahydrate citric acid, and ethylenediamine	Disruption of eDNA structure	eDNA	50.0	[114]
Spermidine, hydrochloric acid, and hydrobromic acid	Chemical dynamic damage	Multiple targets	500.0	[132]
Hydrobromic acid			400.0	[133]
Polyethyleneimine, glutathione, and hemin			100.0	[136]
Citric acid and urea	High ambient temperature		100.0	[115]
Nitro-coronene and branched polyethylenimine			200.0	[148]

Abbreviations: CD, carbon dot; MIC, minimum inhibitory concentration; eDNA, extracellular DNA.

Compared with other antibacterial materials, the structure of CDs is mainly composed of carbon, and its stability is not easily affected by light, temperature, or pH [168]. They are relatively stable in biological systems and cannot be rapidly metabolized or decomposed, making them potentially advantageous for *in vivo* applications [169]. CDs are simpler and more cost-effective to prepare than conventional antimicrobials and less likely to trigger bacterial resistance [26,27,28,170]. CDs bind to bacterial cell membranes, cell walls, nucleic acids, proteins, and enzymes through electrostatic interaction, disrupting their structure and interfering with their function [64,74,171]. Additionally, CDs can be inserted into cell membranes *via* hydrophobic groups on their surfaces, killing bacteria by disrupting membrane integrity [171]. CDs also combat bacterial infections through biochemical damage induced by PTT and PDT under visible light excitation. Therefore, the antimicrobial effects of CDs are not dependent on a single mechanism [161]. Their application in targeted, multimodal synergistic antimicrobial strategies leverages the synergistic interactions between different antimicrobial modes, resulting in enhanced efficiency at lower concentrations and reducing the development of drug-resistant bacteria through a multi-targeting approach [163].

The antibacterial properties of CDs are strongly influenced by their structure [31]. Therefore, by optimizing the structural properties of CDs, their antibacterial properties can be improved, making them more potential for antibacterial applications. The type and density of functional groups formed during carbonization may be different for different precursor materials, such as carboxyl, amino, and hydroxyl groups [172]. The type and number of these functional groups directly affect the surface charge of CDs, hydrophilicity, and interaction with the microbial cell membrane, thus affecting the antimicrobial activity. For example, the surface of CDs generated by the nitrogenous precursor bears more amino groups, which may enhance its binding to the negatively charged bacterial cell membrane and thus improve the antibacterial effect [173]. The variation in the antibacterial properties of CDs derived from different precursor materials offers the potential to synthesize CDs with tailored antibacterial properties. Studying the effects of different precursor materials on the physical-chemical and antibacterial properties of CDs provides an important theoretical basis and guidance for the precise design and optimization of CDs. This direction helps to explore new methods for optimizing the antimicrobial activity of CDs in practical applications.

Despite these advantages, several challenges limit the promotion and clinical application of antibacterial CDs. High concentrations of CDs may have toxicity to nerve cells and mesenchymal cells [174]. The effect of ROS produced by PDT on normal tissues also cannot be ignored. Toxicological studies suggest that while most research supports the safety of CDs, their toxicity varies depending on their synthesis, surface chemistry, and testing methods [175]. The accumulation of CDs may affect its efficacy and safety [176]. To reduce the aggregation risk, one could consider introducing hydrophilic functional groups or polymer chains on the CD surface to enhance their dispersibility or optimizing conditions during preparation to reduce interparticle interactions [177]. Therefore, standardizing the preparation processes, ensuring strict quality control, and improving long-term biosafety are critical steps needed before CDs can be applied clinically.

In complex biological microenvironments, the degradation mechanisms and metabolic pathways of CDs remain poorly understood. Animal studies have shown that CDs are generally excreted by the kidneys, mainly in the urine, but whether they share the same metabolic and excretion pathways in the human body has not been demonstrated [178,179]. Some CDs accumulate in a high-salt environment, and the CD surface may be adsorbed by proteins in an environment containing serum proteins [180]. Advanced techniques, such as gene sequencing and proteomics, to investigate the genes and proteins involved in the degradation of CDs in the body accelerate their clinical application. Moreover, most reported antibacterial CDs are broad-spectrum antibiotics that eradicate pathogenic bacteria while disrupting beneficial microbial communities [181]. Developing CDs with selective antibacterial activity will broaden their potential applications in the gut and other domains. Intelligent, responsive materials are introduced to enhance the antibacterial activity of CDs in response to specific pathogens or environmental stimuli to achieve more precise antibacterial therapy. The broad application prospects of CDs as antibacterial agents in medical device coating, wound dressing, food packaging, and other fields provide diversified development directions for the antibacterial agents of CDs.

Current research on antibacterial CDs focuses primarily on specific infection types or models, making it necessary to broaden validation studies and expand clinical indications [74]. Further attention should be given to evaluating antibacterial effects across various pathogens. Moreover, the clinical application of antibacterial CDs is still in its infancy, with limited large-scale clinical trial data available to support their efficacy and safety. Therefore, enriching disease models and increasing preclinical studies are essential to advancing the clinical use of CDs. Some animal experiments have shown that some CDs have temporary retention in organs, such as the liver and spleen, so there may be a risk of bioaccumulation in long-term use [182,183]. In the future, more long-term studies are needed to evaluate the clearance mechanism and safety *in vivo* fully.

Based on current advanced characterization techniques, researchers now clearly understand the structure of CDs. Additionally, in-depth biological studies have provided a comprehensive understanding of their mechanisms of action *in vitro* and *in vivo*. The utilization of CDs in antimicrobials is anticipated to witness rapid development in the coming years. This review aims to help researchers identify the antimicrobial mechanisms of CDs and guide the development of advanced antimicrobial CDs. CDs with enhanced antibacterial activity and biocompatibility will shortly become first-line drugs against bacterial infections.

## CRediT authorship contribution statement

**Shuaishuai Wang:** Writing – review & editing, Writing – original draft, Conceptualization. **Dapeng Wang:** Writing – review & editing, Writing – original draft, Conceptualization. **Guoliang Wang:** Writing – review & editing. **Minglei Zhang:** Writing – review & editing, Supervision, Conceptualization. **Yirong Sun:** Writing – review & editing, Supervision, Conceptualization. **Jianxun Ding:** Writing – review & editing, Supervision, Project administration, Funding acquisition, Conceptualization.

## Declaration of competing interest

The authors declare that they have no known competing financial interests or personal relationships that could have appeared to influence the work reported in this paper.

## Data Availability

No data was used for the research described in the article.
